# Context-Enhanced Network with Spatial-Aware Graph for Smartphone Screen Defect Detection

**DOI:** 10.3390/s24113430

**Published:** 2024-05-26

**Authors:** Aili Liang, Qishan Wang, Xiaofeng Wu

**Affiliations:** 1 School of Information Science and Technology, Fudan University, Shanghai 200433, China; 21210720031@m.fudan.edu.cn; 2 Academy for Engineering & Technology, Fudan University, Shanghai 200433, China; qswang20@fudan.edu.cn

**Keywords:** defect detection, smartphone screen, attention mechanism, graph reasoning

## Abstract

Interactive devices such as touch screens have gained widespread usage in daily life; this has directed the attention of researchers to the quality of screen glass. Consequently, defect detection in screen glass is essential for improving the quality of smartphone screens. In recent years, defect detection methods based on deep learning have played a crucial role in improving detection accuracy and robustness. However, challenges have arisen in achieving high-performance detection due to the small size, irregular shapes and low contrast of defects. To address these challenges, this paper proposes CE-SGNet, a Context-Enhanced Network with a Spatial-aware Graph, for smartphone screen defect detection. It consists of two novel components: the Adaptive Receptive Field Attention Module (ARFAM) and the Spatial-aware Graph Reasoning Module (SGRM). The ARFAM enhances defect features by adaptively extracting contextual information to capture the most relevant contextual region of defect features. The SGRM constructs a region-to-region graph and encodes region features with spatial relationships. The connections among defect regions are enhanced during the propagation process through a graph attention network. By enriching the feature representations of defect regions, the CE-SGNet can accurately identify and locate defects of various shapes and scales. Experimental results demonstrate that the CE-SGNet achieves outstanding performance on two public datasets.

## 1. Introduction

The development of modern smart devices has brought immense convenience to people’s lives. As a fundamental component of smartphones, the touchscreen has attracted considerable attention regarding its quality. The quality of the touchscreen plays a crucial role in determining the lifespan, user experience and display performance of the smartphone [1]. The smartphone screen is manufactured from glass material, exhibiting favorable mechanical and optical properties. The glass screen for smartphones is produced through a series of specialized processes, including precision Computer Numerical Control (CNC) glass cutting, shaping, polishing, hardening, ultrasonic cleaning, vacuum coating, silk-screen printing, anti-fingerprint oil application, drying, cleaning and inspection [2]. The entire process imposes strict requirements on the production environment. However, during the processes of production and transportation, various defects invariably arise, including scratches, cracks, breakages, spots and so on. Such defects affect the aesthetic appeal of smartphone screens and lead to adverse effects on their production quality. Therefore, defect detection on smartphone screens is a pivotal step in the industrial process, aiming to avoid process waste, reduce production costs and ensure product quality.

Currently, smartphone screen defects are primarily detected through traditional manual inspection or machine vision methods, both of which have certain limitations. Traditional manual inspection, conducted by experienced inspectors under strong lighting, consumes significant time and energy, and may lead to vision impairment. This approach struggles to meet the demands of large-scale industrial production, hindering automation and intelligence in the production process. On the other hand, machine vision methods rely on handcrafted feature-extraction techniques. Subsequently, traditional machine learning algorithms are employed for defect detection [3,4]. The machine vision methods overcome the drawbacks of traditional manual inspection, but their reliance on manual rules or thresholds often results in an elevated occurrence of false negatives, particularly for small defects. Additionally, they have limitations in stability, accuracy and adaptability to varying environments.

With the rapid progress in computer vision technologies, the emergence of deep learning, notably convolutional neural networks (CNNs), has opened up new avenues for smartphone screen defect detection. CNNs extract features from original data without handcrafted feature extractors, offering expressive feature representation. The classical object detectors based on CNNs can be broadly categorized into two types: two-stage detectors like Faster RCNN [5] and Cascade RCNN [6], and one-stage detectors such as YOLO [7], RetinaNet [8] and FCOS [9]. The primary distinction lies in the methodology: two-stage detectors generate region proposals (RPs) for further classification and regression, while one-stage detectors predict classification and position directly from the features. In contrast, two-stage detectors typically achieve higher detection accuracy.

**Figure 1:**
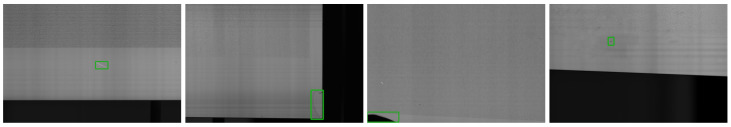
Some examples of smartphone screen defect images. The categories of defects include scratch, crack, breakage and spot.

General detectors are often designed for natural images and may not achieve optimal performance when applied to the specific task of smartphone screen defect detection. Compared to natural images, smartphone screen defect images exhibit unique characteristics. Firstly, screen defects tend to be small and irregular, such as spots and scratches, which leads to limited feature information and a high false-negative rate. Secondly, defects like scratches and cracks exhibit minimal color contrast against the screen background. Smartphone screen images are typically captured by high-resolution line-scan cameras, with a black background to minimize interference from ambient light. Despite these efforts, the features of defects still do not stand out prominently, presenting a significant challenge to detection. Some examples are shown in Figure 1. These unique characteristics emphasize the necessity for tailored methods to improve the performance of defect detection on smartphone screens.

Several detection methods for screen defects have been proposed to enhance defect detection performance. Wang et al. [10] applied Faster RCNN to detect smartphone screen defects. Faced with the challenge of a limited number of samples, they employed Boundary Equilibrium Generative Adversarial Networks (BEGAN) to generate and enhance defect data, achieving better performance compared to machine vision methods. Chen et al. [11] proposed a defect detection network based on the fusion of Faster RCNN, and they employed U-Net [12] and ResNet50 [13] for feature extraction. U-Net is employed to capture detailed information about defects, while ResNet50 extracts high-level semantic information. The combination of both can obtain more distinctive feature information, effectively detecting three common surface defects on screens. However, the fusion of two feature-extraction networks led to higher complexity of the model. Some works have introduced attention mechanisms to enhance the network’s focus on defect features. Zhang et al. [14] proposed a category-aware network for detecting edge cracks on smartphone screens. They employed multiple parallel feature-extraction branches to capture features at different scales and adjusted channel weights through a category-aware attention mechanism. Zhu et al. [15] introduced a channel attention mechanism for detecting scratches on smartphone screens. They used high-frequency information and local cross-channel interactions to represent weighted defect features, reducing feature loss during global average pooling and enhancing the feature of tiny defects. Additionally, they employed Region of Interest (RoI) Align to handle scratches with extreme aspect ratios. However, these methods [14,15] only focus on channel attention, neglecting spatial attention. Attention to spatial positions is crucial because it determines the accuracy of localization in defect detection.

In the task of smartphone screen defect detection, previous defect detection methods face two prevalent challenges: (1) In defect images, small defects lead to an imbalance in the distribution of normal and defective pixels, which causes the network to excessively focus on the background, resulting in insufficient attention to information of defect features. (2) Most defect detection methods focus on individual regions, neglecting the relationship among defects and the implicit prior knowledge embedded in the image.

To overcome the two challenges, this paper proposes a defect detection method including an attention mechanism and spatial-aware graph reasoning, named CE-SGNet. CE-SGNet introduces two pivotal modules: the Adaptive Receptive Field Attention Module (ARFAM) and the Spatial-aware Graph Reasoning Module (SGRM). The ARFAM is applied to perform attention-weighting on the fused multi-scale features by employing channel attention and spatial selection attention. It dynamically captures diverse contextual information necessary for various defects, enhancing the expressive capability of defect features. In the SGRM, proposals are employed to construct a region-to-region relation graph under category semantic supervision. The CE-SGNet learns adjacency relationships among proposals autonomously, and propagates the updated features globally. Finally, the enhanced feature of each region is concatenated with the original RoI feature for classification and localization.

In summary, the major contributions of this paper can be summarized as follows:

1. A novel attention module called ARFAM is proposed, which integrates channel attention and spatial selection attention. The channel attention focuses on the importance of different channel features, while the spatial selection attention dynamically adjusts the requirements of diverse contextual information for different defect types, thereby enhancing the model’s ability to express features.
2. The SGRM introduces the Spatial-aware Graph Attention Network (SGAT) to model the relationships among regions. A learnable relation graph is constructed to facilitate adaptive reasoning for global features, effectively propagating spatial and contextual information.
3. Experiments conducted on a real-world dataset of smartphone screen defects illustrate the effectiveness of the proposed method in defect detection. Furthermore, our method is evaluated on a public defect dataset to demonstrate its generalization capability.

The remainder of this paper is organized as follows. Section 2 describes the related works. Section 3 presents the proposed method. Section 4 shows the experimental results, analysis of every module, visualization study, generalization study, model complexity and time-efficiency evaluation. Section 5 discusses the limitations of the proposed method and the future research directions. Section 6 presents the conclusion.

## 2. Related Works

### 2.1. General Object Detectors

Object detection is a crucial task in computer vision. In recent years, there has been significant progress in object detection methods based on deep learning. Generally, these methods can be divided into two categories: two-stage detectors and one-stage detectors. Two-stage detectors, represented by the RCNN series, such as Faster RCNN [5] and Cascade RCNN [6], first propose candidate regions and then perform classification/regression. Taking Faster RCNN [5] as an example, it generates RPs through the Region Proposal Network (RPN) firstly and subsequently employs RoI pooling to map these proposals of various sizes to a fixed-size feature map for accurate classification and localization. Cascade RCNN [6] improves detection performance by cascading multiple detection heads to refine proposals. Compared to two-stage detectors, one-stage detectors directly predict classification and location without RoI proposals. For example, YOLO [7] directly predicts classification and location by generating dense anchor boxes on the input image. RetinaNet [8] introduces a loss function called Focal Loss to address class imbalance in object detection. FCOS [9] is an anchor-free detector that predicts the points of objects to form bounding boxes. In comparison, two-stage detectors achieve higher detection accuracy through refined design, which makes them more suitable for high-accuracy scenarios.

In recent years, there has been an increase in methods utilizing Transformer for detection. Carion et al. [16] introduced DETR, a pioneering method that applies Transformer structure to object detection tasks. DETR formulates object detection as a set prediction problem, achieving end-to-end detection. To solve the slow convergence and poor performance in detecting small objects in DETR, Deformable DETR [17] introduced a deformable attention mechanism to flexibly adapt to the deformation and changes in the object shapes. However, these detectors often require significant computational resources and are easy to overfit, particularly when dealing with small datasets such as defect detection datasets.

### 2.2. Attention Mechanisms

Attention mechanisms simulate the function of the human visual system, enabling neural networks to focus on crucial parts of an image, thus improving the model’s performance and generalization capability. Generally, attention mechanisms include channel attention, spatial attention and self-attention. SENet [18] is a classic attention mechanism network that learns channel weights through global pooling layers, enhancing the importance of different channels in feature maps. Expanding on the foundation of channel attention, CBAM [19] introduces spatial attention, dynamically adjusting weights for both channels and spatial positions in feature maps to enhance overall model performance. SKNet [20] introduces a selective kernel module responsible for selectively applying convolutional kernels of different sizes on each feature map, better capturing multi-scale information in the image. Additionally, the self-attention mechanism in Transformer [21] allows each input position to interact with others, generating corresponding attention weights, which enables the model to capture long-range dependencies in sequential data. However, this method operates on the entire feature map, demanding significant computational resources. Our attention module draws inspiration from the CBAM structure, but we refine the spatial attention module through extracting adaptive contextual information for various defects. This makes it more suitable for smartphone screen defects.

### 2.3. Graph Neural Networks

Extensive research has been carried out in diverse visual domains to explore relation reasoning through objects. In recent works, objects and their relationships are frequently represented as graph structures, leveraging Graph Neural Networks (GNNs) [22] for their flexibility in modeling dependencies between nodes, particularly in scenarios involving irregular data structures. Graph Convolutional Networks (GCNs) [23] extend CNNs to graph-structured data, enabling nodes to focus on semantic or spatial neighborhood features. Several works have applied graph structures to defect detection. In work [24], structured knowledge graphs are integrated into a learning system to enhance the accuracy and reliability of additive manufacturing defect diagnosis. Wang et al. [25] addressed the issues of inter-class similarity and intra-class variability in defect detection by constructing a class-balanced graph. They applied this approach in domains such as steel and textiles. Zhai et al. [26] focus on insulator defect detection, constructing both a multi-geometry reasoning network and an appearance-geometry reasoning network to extract appearance and geometry features of defect samples, respectively. However, these works ignore the spatial relationships between defect regions and implicit information in images. Therefore, we construct a region-to-region graph and employ Graph Attention Networks (GATs) [27] for the propagation and updating of region features.

## 3. Method

### 3.1. Overview

**Figure 2:**
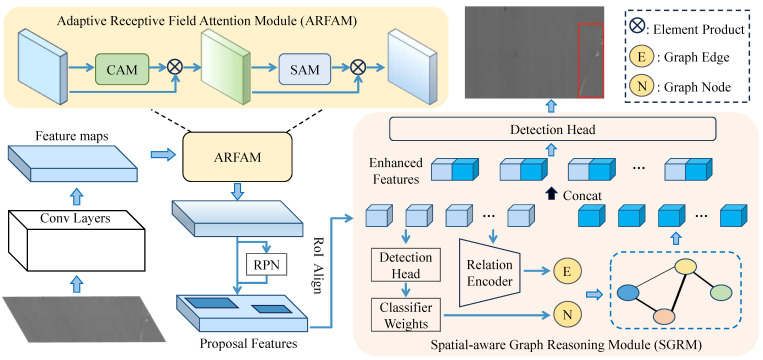
The overall architecture of proposed CE-SG defect detection network. It consists of two innovative modules: the Adaptive Receptive Field Attention Module and the Spatial-aware Graph Reasoning Module.

The proposed architecture is illustrated in Figure 2. The two-stage detector Faster RCNN [5] with FPN [28] is employed as the baseline. Initially, the feature maps of defect image are extracted through the backbone, and then passed through the FPN for feature fusion. The fused feature maps are processed by the ARFAM for adaptive receptive field attention weighting. After extracting RPs through the RPN and RoI Align, the SGRM utilizes these proposals to construct a region-to-region graph guided by the previous classification results. The graph node embeddings of different regions are evolved and propagated by SGAT. Finally, the enhanced feature of each region is concatenated with the original feature for classification and localization.

### 3.2. Adaptive Receptive Field Attention Module

**Figure 3:**
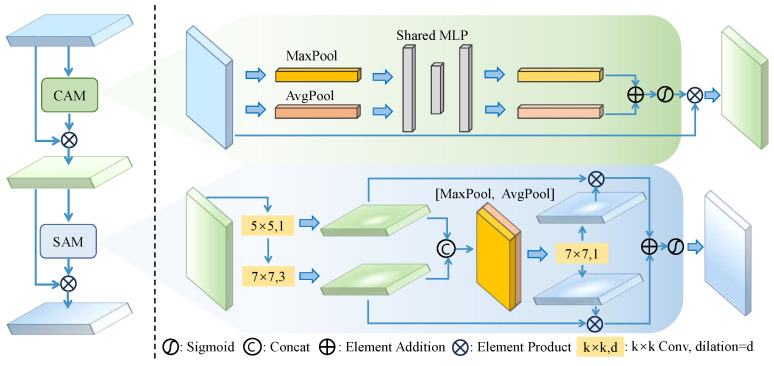
Illustration of the Adaptive Receptive Field Attention Module.

In the task of smartphone screen defect detection, there are numerous defects with low contrast and poor saliency. During the detection process, these defects may be considered background noise, leading to missed detection. Moreover, the pixel area of defects in the image is much smaller than that of the background, leading to the network paying excessive attention to the background instead of foreground. In computer vision, attention mechanisms are widely applied, effectively improving the performance of networks. Therefore, we propose the ARFAM to enhance defects’ features and obtain wide contextual information, as illustrated in Figure 3.

The ARFAM consists of a Channel Attention Module (CAM) and a Spatial Attention Module (SAM). The CAM learns channel feature weights, enhancing the features of relevant channels based on these weights while suppressing irrelevant channel features. Firstly, the input feature map $F_{in}$ undergoes separate weighting through both global average pooling (GAP) and global maximum pooling (GMP). Afterward, the features generated by the MLP layers are subjected to element-wise summation, followed by sigmoid activation to produce the channel attention map $M_{c}\left(F_{in}\right)$. The formula is as follows:

(1) $$M_{c}\left(F_{in}\right)=\sigma \left(MLP\left(GAP\left(F_{in}\right)\right)+MLP\left(GMP\left(F_{in}\right)\right)\right),$$

where σ is the sigmoid activation function. MLP is a two-layer neural network with the first layer having $N_{c}/r$ neurons, activated by ReLU, and the second layer having $N_{c}$ neurons, where *r* is the reduction ratio and $N_{c}$ is the number of neurons. The channel attention feature map $F_{c}$ is combined with $F_{in}$ as $F_{c}=F_{in}\times M_{c}\left(F_{in}\right)$, generating the input features for the SAM.

The SAM adaptively adjusts the receptive field of the channel-weighted feature map, effectively handling diverse contextual information for different defects. In defect detection, extensive contextual information can help the network localize defects effectively, and the range of contextual information varies for different types of defects. Therefore, it is necessary for the network to dynamically adapt the range of defect contextual information. According to [29], large kernel convolution can increase the receptive field of the image, thereby obtaining more extensive contextual information. Therefore, a series of depth-wise convolutions with different kernels are applied to construct larger kernel convolutions, which can control the range of contextual information for different types of defects. The receptive field of the i-th layer $RF_{i}$ can be represented as

(2) $$RF_{i}=RF_{i-1}+d_{i}\left(k_{i}-1\right),$$

where $d_{i}$ is the dilation rate and $k_{i}$ is the kernel size. The feature maps from different convolutional kernels can be represented as:

(3) $$F_{s1}=f_{1}^{dw}\left(F_{c}\right),F_{s2}=f_{2}^{dw}\left(F_{s1}\right),$$

where $f_{i}^{dw}\left(\cdot \right)$ are depth-wise convolutions. Then they are concatenated as $F^{\prime }=\left[F_{s1};F_{s2}\right]$, and $F^{\prime }$ is spatially selected through channel-based maximum pooling and average pooling. Finally, a convolution layer is applied to generate spatial selection weighting coefficients from the pooled feature maps:

(4) $$C_{s}=f^{7\times 7}\left(\left[AvePool\left(F^{\prime }\right);MaxPool\left(F^{\prime }\right)\right]\right).$$

The spatial attention map is generated by weighting each receptive field feature map, followed by sigmoid activation:

(5) $$M_{s}\left(F\right)=\sigma \left(\sum_{i}C_{si}\times F_{si}\right).$$

The SAM enables adaptive selection of contextual information for different defects, enabling the network to capture spatial contextual regions most relevant to the defect features.

The ARFAM dynamically captures the long-range dependency of defect features through adjusting the range of the receptive field, enhances the network’s attention to defect features and enables more accurate localization. Generally, attention mechanisms are employed in the convolution layers for extracting features. However, we extrapolate that it may still introduce noise during feature fusion due to the inconsistency of features across different scales, thereby affecting detection results. Therefore, the ARFAM is inserted after FPN in this paper and adaptive weighting is conducted individually for feature maps at different scales. The experiments in Section 4.3.3 validate its effectiveness.

### 3.3. Spatial-Aware Graph Reasoning Module

**Figure 4:**
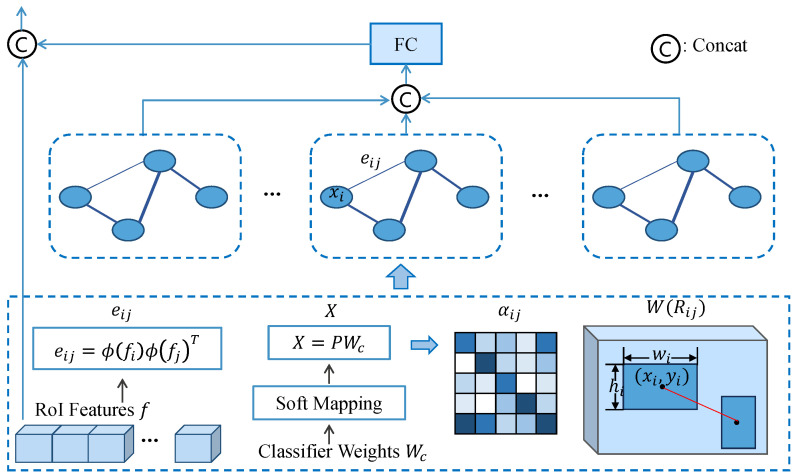
Flowchart of the Spatial-aware Graph Reasoning Module.

Smartphone screen defects, caused by mechanical reasons, often tend to occur in specific locations with distinct types or textures. For instance, cracks often occur in edge regions, and some defects are typically incomplete. In addition, downsampling in CNNs may lead to the loss of features in small defects, posing challenges for detection. Previous works usually relied on every RP for classification and regression. However, independent RPs contain limited feature information, which poses challenges for detection. We hope to leverage information within the images to enhance low-quality features. In order to enhance the representation of various defects and their interrelations, a graph network is proposed to model the spatial relationships between defect regions and enhance the RoI features. Figure 4 shows the flowchart of the SGRM.

#### 3.3.1. Spatial-Aware Graph Building

In the initial graph representation as G=<V,E>, where V represents node features, and each node represents each RP, each edge $e_{i,j}\in E$ encodes the relationship between two regions. Some works use handcraft linguistic knowledge to build graphs, which may ignore the spatial relationships. Due to the gap between visual and language domains, we refrain from introducing explicit linguistic knowledge. Instead, the relationship matrix between regions is learned through RoI features *F*, i.e., $e_{ij}=\phi \left(f_{i}\right)\phi {\left(f_{j}\right)}^{T}$, where ϕ(·) denotes a non-linear function. In this paper, ϕ(·) is obtained through two fully connected layers with ReLU activation, and $f_{i}\in F$ represents the feature of RP *i*.

The regions proposed by RoI Align are independent of each other and exhibit low correlation between regions. Furthermore, these proposals may not accurately depict defect information. When a region proposal is a negative sample, it can propagate incorrect information. Hence, utilizing proposal features directly as graph node feature embeddings not only affects detection results but also introduces noise. By guiding the regions with advanced semantic information, the initial feature embeddings of graph node can be aligned more closely with the actual region features. This facilitates a more accurate modeling of relationships among defects and enhances the network’s learning capability. Inspired by the work in [30], the previous classifier weights $W_{c}\in R^{C\times (D+1)}$ are employed, which contain advanced semantic information in the graph node embedding. The weights can be obtained by copying the parameters with the bias from the previous classification layer, updated throughout network training. To build a region-to-region graph, it is necessary to map category embeddings to region feature representations of nodes. While the previous classifier scores could achieve this mapping, incorrect classification results could lead to erroneous node feature representations. Therefore, we propose a soft mapping denoted by

(6) $$p_{i}=\frac{\exp(s_{ij})}{\sum_{j}\exp\left(s_{ij}\right)},$$

where $s_{ij}$ represents the score of RP *i* being classified into category *j*, $p_{i}\in P\in R^{N\times C}$ is the mapping weight coefficient. The node feature embedding can be computed as $X=PW_{c}$, where $X\in R^{N\times (D+1)}$, *N* is the number of nodes, *C* is the number of categories, and *D* is the dimension of the graph node features. Employing classification weights as supervision can improve the correlation among similar defects and accentuate the distinctions between different defect categories. This facilitates more accurate propagation of node feature information.

#### 3.3.2. Spatial-Aware Graph Reasoning

To construct a graph with rich spatial information, the relative positional relationship between defects is crucial. Therefore, the spatial relationship between region *i* and region *j* is formulated as

(7) $$R_{ij}={\left(log\left(\frac{x_{i}-x_{j}}{w_{i}}\right),log\left(\frac{y_{i}-y_{j}}{h_{i}}\right),log\left(\frac{w_{j}}{w_{i}}\right),log\left(\frac{h_{j}}{h_{i}}\right)\right)}^{T},$$

where $\left(x_{i},y_{i}\right)$ represents the central coordinates of region *i*, and $\left(w_{i},h_{i}\right)$ denotes the width and height of region *i*, which are normalized. The feature will be mapped to a high-dimensional space to encode spatial information.

The SGAT is employed for updating the graph, which allows the selection of more relevant nodes based on their importance, implicitly assigning different weights to nodes in the neighborhood. The attention mechanism is used to autonomously learn and optimize the connection relationships between graph nodes. The update formula for nodes is as follows:

(8) $$X^{l+1}=\sigma \left(\sum_{j\in N_{i}}\alpha_{ij}W^{l}\left(R_{ij}\right)X^{l}\right),$$

where $X^{l}$ represents the node feature embedding for the *l*-th layer of the SGAT, $W^{l}\left(R_{ij}\right)$ is a learnable weight matrix encoding spatial position information of the nodes. $\alpha_{ij}$ represents the normalized spatial relation coefficient, computed as the weighted sum of node feature representations:

(9) $$\alpha_{ij}=\frac{\exp(\mathrm{LeakyReLU}(a^{T}[WX_{i}||WX_{j}]))}{\sum_{l\in N_{i}}\exp(\mathrm{LeakyReLU}(a^{T}[WX_{i}||WX_{l}]))},$$

where $X=\left\{X_{1},X_{2},\cdots ,X_{N}\right\}$, *N* is the number of regions, || denotes the concatenation operator and $a^{T}$ represents the attention coefficients. Multi-head attention layers are used to propagate spatial relations among nodes, and the propagation process of the network is defined as:

(10) $$F_{enc}=\overset{K}{\underset{k=1}{\left|\right|}}\sigma \left(\sum_{j\in N_{i}}\alpha_{ij}^{k}W^{l}\left(R_{ij}\right)X^{l}\right),$$

where *K* is the number of multi-head attention layers. Finally, the enhanced features $F_{enc}$ generated by SGAT are dimensionally mapped and concatenated with the original features *F* for classification and localization in the detection head.

In the SGRN, advanced semantic features and implicit information in the images guide the construction of the graph and the propagation of node features. In this process, proposal features with semantic similarities strengthen each other, while those with significant differences are suppressed, reducing the positional offset between bounding boxes and ground truth. It is beneficial to capture complex relationships between regions globally, aiding the network in understanding more intricate scenes, such as dense distributions.

## 4. Experimental Results and Analysis

### 4.1. Experimental Setup

#### 4.1.1. Datasets

The smartphone screen glass defect (SSGD) dataset [31] is a popular public dataset designed for smartphone screen glass defect detection tasks. It comprises 2504 images, each with dimensions of 1500 × 1000 pixels. It includes seven common defect categories: crack, broken, spot, scratch, light-leakage, blot and broken membrane. All images are captured from real-world scenarios, providing a comprehensive representation of potential screen defects with a certain level of complexity. The dataset is challenging, and some examples are shown in Figure 1. The dataset comprises two sub-datasets, namely LB101 and LB201, collected from different working platforms. These sub-datasets exhibit variations in defect sample distribution, types of screens and screen background colors. We validate the effectiveness of the proposed method on each of the two subsets separately. The dataset is randomly separated into a training and a testing set with a ratio of 4:1.

To prove the generalization of the proposed method, the printed circuit board (PCB) dataset released by Peking University is introduced. It consists of 693 images with 6 types of defects: missing hole, mouse bite, open circuit, short, spur and spurious copper. The image resolutions range from 3056 × 2464 to 2904 × 1521. The dataset is randomly separated into a training and a testing set with a ratio of 4:1.

#### 4.1.2. Evaluation Metrics

In the defect detection task, precision and recall are crucial metrics. The Precision–Recall (PR) curve illustrates the relationship between precision and recall, and the Average Precision (AP) is calculated as the area under this curve. AP combines precision and recall to evaluate the overall detection performance of the model across different thresholds. The mean Average Precision (mAP) is the average of AP values across all categories. We employ evaluation metrics following the COCO standard, including AP, AP_50_, AP_75_, AP_*s*_, AP_*m*_ and AP_*l*_. Here, AP represents the average precision at IoU thresholds ranging from 0.5 to 0.95 with intervals of 0.05. AP_50_ and AP_75_ represents average precision at IoU thresholds of 0.5 and 0.75, respectively. AP_*s*_, AP_*m*_ and AP_*l*_ represent average detection precision for small (area $<32^{2}$), medium ($32^{2}<$ area $<96^{2}$) and large (area $>96^{2}$) objects, respectively. These metrics comprehensively evaluate the defect detection performance in general scenes, high-precision localization scenes and multi-scale detection capability.

#### 4.1.3. Implementation Details

The CE-SGNet is constructed of the PyTorch framework and the experiments are performed on 8 NVIDIA GeForce RTX 3090 GPUs. Stochastic Gradient Descent (SGD) with a momentum of 0.9 and weight decay of 0.05 serves as the optimizer. The initial learning rate is set to 0.02, with a batch size of 8. The training strategy follows a 1× schedule (12 epochs), with learning rate reductions by a factor of 10 at the 8th and 11th epochs to prevent overfitting due to excessively large learning rates in the later iteration of training. All models are initialized with weights pre-trained on the COCO dataset, ensuring a solid starting point for defect detection. Random flips and multi-scale training are applied to images during training. To avoid potential experimental bias, the final evaluation metrics are obtained by averaging the results from five experiments.

### 4.2. Comparative Experiments

To assess the performance of the proposed method, the CE-SGNet is compared with nine representative detectors. These detectors include: two-stage typical detectors Faster RCNN [5] and Cascade RCNN [6], one-stage anchor-based detector RetinaNet [8], GFL [32] and YOLOv5 [33], anchor-free detector FCOS [9] and ATSS [34], transformer-based detectors DETR [16] and Deformable DETR [17]. YOLOv5 stands out as one of the most widely used one-stage detectors in the field of defect detection. It employs CSPDarkNet53 as the backbone and is trained for 100 epochs. To avoid parameter redundancy, the lightest model YOLOv5-s is selected for the experiment. DETR and Deformable DETR represent typical transformer-based detectors, utilized for performance comparison with CNN-based detectors. They are trained for 50 epochs using the Adam optimizer. All the other models use ResNet50 as the backbone and FPN as the neck. All methods are implemented based on MMDetection with official hyperparameter settings. The same preprocessing operations are applied, including multi-scale data augmentation and dataset splitting, to ensure a fair comparison.

The detailed results on the SSGD dataset are presented in Table 1. It is observed that the proposed method outperforms other methods in most metrics, demonstrating superior performance. Compared to the baseline model, the CE-SGNet achieves better performance in all metrics, which indicates the effectiveness of both ARFAM and SGRM. ARFAM enables the model to focus more on defects and their contextual information, enhancing the model’s ability to learn defect features. Subsequently, due to the improved localization accuracy achieved by SGRM, CE-SGNet demonstrates robust performance across various IoU thresholds, making it suitable for both general scenarios and high-precision localization scenarios. In terms of multi-scale detection capability, it also achieves excellent performance. The performance improvement in both small and medium defects are higher than that in large defects, as CE-SGNet enriches the limited features in small and medium defects, thereby enhancing performance. In summary, the CE-SGNet improves detection performance across the board and does not sacrifice the performance of one metric to improve another metric.

Furthermore, it is observed that two-stage methods generally outperform one-stage detectors in AP. This is attributed to the effectiveness of RPN in distinguishing foreground and background, reducing false positives. One-stage detectors, without rough filtering by RPN, exhibit lower accuracy compared to two-stage detectors. Furthermore, there exists a notable performance disparity between transformer-based models and CNN-based methods. This indicates that transformer-based models encounter challenges in demonstrating their advantages, especially when handling small datasets. The comprehensive analysis of results highlights the efficacy of the CE-SGNet in smartphone screen defect detection.

### 4.3. Performance Analysis of Modules

In order to validate the effectiveness of the proposed modules, we conduct comprehensive experiments on the SSGD-LB101 dataset, utilizing metrics such as AP and AP_50_ to evaluate the detection performance of different modules.

#### 4.3.1. Analysis of Each Module

The results of the effectiveness of each module are shown in Table 2. As observed, each module played an effective role, improving performance independently. The ARFAM enhances the sensitivity of the detection model, improving the model’s perception and recognition capabilities for detecting defects. Although the SGRM partially improves performance through constructing relationships between regions, the RoI features become more accurate after ARFAM extracting extensive contextual information, thereby resulting in higher performance improvement. The combined action of the two modules results in an increase of AP to 24.0 and AP_50_ to 51.2, showing improvements of 3.7 AP and 3.9 AP_50_, respectively. This indicates the effectiveness of each proposed module and the combined action of both does not have a negative impact on performance.

#### 4.3.2. Analysis of the Attention Mechanisms

The compared attention mechanisms include SENet [18], CBAM [19], ARFAM with only channel attention (CAM), ARFAM with only spatial attention (SAM) and the proposed ARFAM. The computational complexity (Giga Floating Point Operations per Second, GFLOPs) and the total number of trainable parameters in the model (Params) are also employed for evaluating the efficiency of models. For a fair comparison, all other parameters and experimental settings remain consistent, with the only difference being the type of attention mechanism. As shown in Table 3, the results demonstrate that the combination of channel attention and spatial attention can effectively improve the detection performance. CAM, which adds a global maximum pooling layer compared to SENet [18], can effectively extract the most prominent features of defects, aligning with conclusions from CBAM [19]. In comparison to CBAM [19], the ARFAM increases 0.7 AP and 1.3 AP_50_ respectively, without a significant increase in computational resources or model parameters. This indicates that the ARFAM can fully extract the most relevant contextual information of defects by adaptively selecting receptive fields.

#### 4.3.3. Analysis of Different Positions of the ARFAM

We also conduct an analysis of the different positions of ARFAM. “ARFAM+FPN” represents ARFAM inserted before FPN, “FPN+ARFAM” represents ARFAM inserted after FPN without shared parameters and “FPN+ARFAM-shared” represents ARFAM inserted after FPN with shared parameters. According to the experimental results in Table 4, “ARFAM+FPN” reduces AP by 0.8 and AP_50_ by 2.4 compared to “FPN+ARFAM”. If the ARFAM is inserted before FPN, it may introduce noise during the feature fusion process. The defect features on different feature maps may be inconsistent, which affects the stability of model learning. Additionally, “ARFAM+FPN” leads to an excessive increase in model parameters, causing parameter redundancy and decreasing accuracy. Shared parameters tend to promote consistent attention across different scales, but the focus positions often vary across scales in practice. By inserting ARFAM after FPN, irrelevant feature interference before fusion is reduced, resulting in more accurate candidate boxes generated by RPN.

#### 4.3.4. Analysis of the SGRM

**Figure 5:**
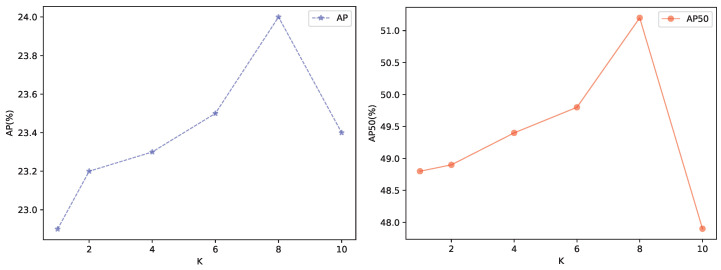
Comparison results of different numbers of attention heads.

Finally, we analyze different mapping ways for node embedding. According to the results shown in Table 5, the soft mapping increases 0.6 AP and 0.8 AP_50_ compared to the hard mapping. Moreover, it does not require additional computational costs or an increase in model parameters. However, hard mapping may increase the error rate if the initial classification results are incorrect. In addition, We also conduct an analysis with different numbers of the head in SGAT, setting k to 1, 2, 4, 6, 8, 10, as shown in Figure 5. Based on the results, the detection performance continuously improves with an increase in the number of heads until a sudden drop is observed when there are 10 heads in the multi-head attention mechanism. Considering both AP and AP_50_, the number of heads is set to 8 in this paper.

### 4.4. Visualization of Results

**Figure 6:**
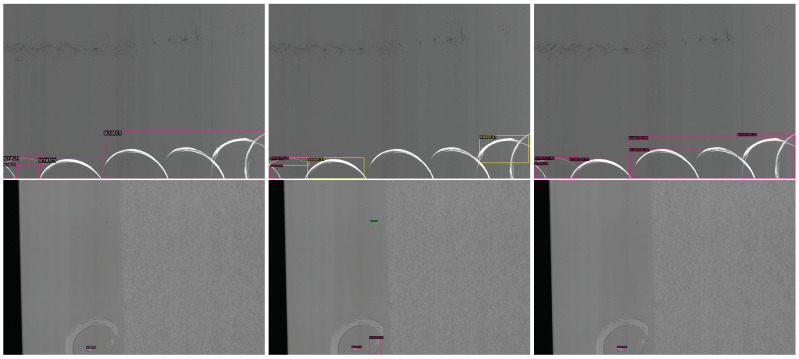

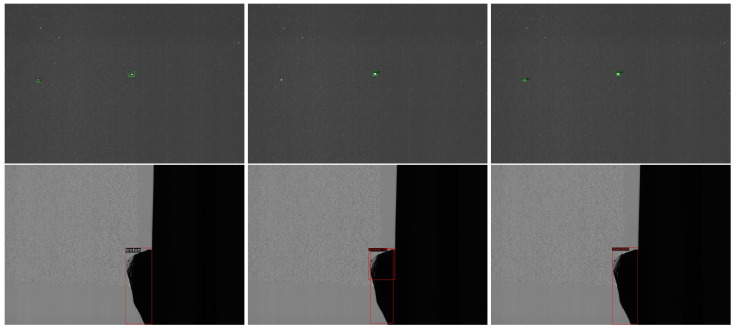
Examples of visualization results on the SSGD dataset. The first column is the ground truth, the second column is the results of the baseline model and the third column is the results of our model.

To visually show the effectiveness of the CE-SGNet, Figure 6 presents qualitative comparison results with the baseline model. It is observed that the CE-SGNet is more effective than the baseline model in detecting difficult defects, such as small spots. Moreover, the defect regions detected via CE-SGNet are more accurately aligned with the ground truth. The results indicate that the CE-SGNet has the capability to reduce both false positives and false negatives, improving overall detection performance. The ARFAM enhances contextual information of defects, which is beneficial for identifying defects accurately. Moreover, the SGRM strengthens the interdependence between defects, improving localization accuracy.

**Figure 7:**
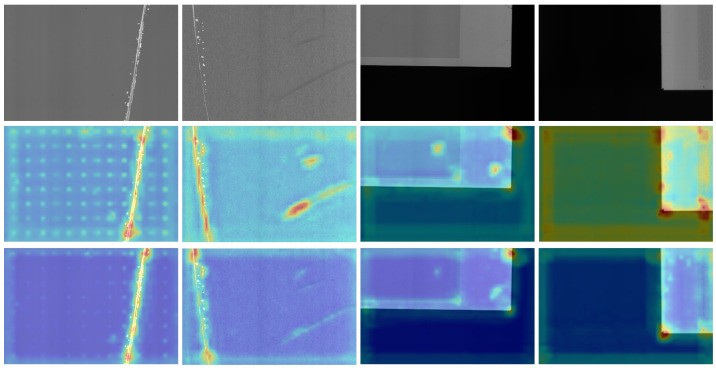
Examples of feature map visualization results on the SSGD dataset. The first row is original images, the second row is feature maps of baseline and the third row is feature maps of CE-SGNet.

We also visualize the feature maps to provide an intuitive understanding of the defect regions attended by ARFAM. The results in Figure 7 demonstrate that the ARFAM effectively suppresses background feature information and enhances contextual information. For instance, in the first and second columns of Figure 7, it successfully suppresses spots or blots in the background which are not defects. It is beneficial for RPN to perform more accurate classification between foreground and background. Moreover, our CE-SGNet adapts contextual information based on defect size, as shown in the third and fourth columns of Figure 7. Compared to the baseline model, the CE-SGNet identifies regions closer to the ground truth, demonstrating its effectiveness in improving detection accuracy.

### 4.5. Generalization Study

To verify the generalization capability of the CE-SGNet, we conduct generalization experiments on the PCB dataset. The CE-SGNet is compared with Faster RCNN [5], Cascade RCNN [6], RetinaNet [8], FCOS [9], ATSS [34], GFL [32], YOLOv5-s [33], DETR [16] and Deformable DETR [17]. The results are shown in Table 6. It can be observed that the proposed method achieves 53.8 AP, achieving optimal performance. In terms of multi-scale detection capability, the proposed method achieves the highest accuracy in AP_*s*_, AP_*m*_ and AP_*l*_ compared to other methods. This shows that the CE-SGNet can be applied to scenarios with different defect sizes, especially for detecting small defects. The experimental results demonstrate that the CE-SGNet exhibits excellent generalization performance.

### 4.6. Model Complexity and Time-Efficiency Evaluation

To evaluate the complexity and efficiency of the CE-SGNet, GFLOPs, Params and frames per second (FPS) are employed as the evaluation metrics. GFLOPs is used to evaluate the computational complexity, Params is used to evaluate the model size and memory usage, while FPS is used to evaluate the real-time efficiency. The comparative results are shown in Table 7. It is observed that CE-SGNet exhibits higher GFLOPs at 347.37 compared to the baseline model, due to the inclusion of two novel components. In terms of model parameters, Cascade RCNN consumes the highest memory resources. In contrast, our CE-SGNet, while requiring additional computational resources and a slightly larger model size, obtains a significant improvement in detection performance. However, the introduction of SGAT slows down CE-SGNet’s inference speed, impacting its real-time performance.

## 5. Discussion

In this paper, we propose the CE-SGNet based on two-stage detectors for smartphone screen defect detection. While the CE-SGNet achieves excellent performance, there are still some limitations. Firstly, as shown in Table 7, our CE-SGNet consumes more computational resources and has a complex model structure. In terms of inference speed, it may face challenges in real-time detection scenarios. Future research can focus on accelerating the structure of CE-SGNet through appropriate light-weighting methods to improve the real-time detection capability of our model. Secondly, due to the unique optical properties of screen glass, the color of defects is similar to that of the background. This means that not all objects identified as defects based on visual perception are real defects. Most detection methods improve detection performance by enhancing model sensitivity, which also leads to an increase in false positives. In the future, exploring the fusion of multimodal knowledge, such as language and image, could provide a more comprehensive understanding of industrial scenarios.

## 6. Conclusions

In our research, a context-enhanced spatially aware defect detection network that effectively detects defects on smartphone screens is proposed. We introduce two modules: the ARFAM and the SGRM. The ARFAM, composed of a channel attention module and a spatial attention module, obtains wide contextual information and suppresses background features, effectively detecting low-contrast tiny defects. The SGRM, constructs a region-to-region graph to strengthen the spatial relationship between defects and reduce false negatives. Experimental results both on the SSGD dataset and the PCB dataset illustrate that the proposed method demonstrates significant improvement in detection accuracy, highlighting its considerable potential for practical applications.

## Figures and Tables

**Table 1:** Comparison results of different methods on the SSGD dataset. Bold data denote the best performance. Underlined data denote the second best performance.

Model	Backbone	LB101						LB201					
		AP	AP_50_	AP_75_	AP_*s*_	AP_*m*_	AP_*l*_	AP	AP_50_	AP_75_	AP_*s*_	AP_*m*_	AP_*l*_
Faster RCNN [5]	ResNet50	20.3	47.3	17.0	13.4	28.2	34.4	20.4	43.8	16.4	20.7	18.7	22.1
Cascade RCNN [6]	ResNet50	21.0	44.2	16.8	15.1	25.3	41.0	22.2	47.1	18.4	22.3	19.3	**23.8**
RetinaNet [8]	ResNet50	16.5	35.5	13.8	12.2	24.6	29.4	17.8	39.3	13.0	22.5	16.7	17.3
FCOS [9]	ResNet50	18.2	43.1	13.2	13.4	24.1	26.4	19.9	44.1	15.5	19.3	20.2	16.9
ATSS [34]	ResNet50	18.3	42.0	13.9	15.8	25.2	26.2	21.5	44.6	17.5	21.9	19.5	23.3
GFL [32]	ResNet50	18.1	40.6	14.6	14.1	25.1	28.0	21.7	44.7	18.4	20.9	20.4	23.5
YOLOv5-s [33]	CSPDarkNet53	16.4	42.7	9.3	8.3	23.3	25.1	15.2	34.1	12.4	8.7	17.0	14.7
DETR [16]	ResNet50	11.5	23.0	10.1	3.5	12.3	27.4	11.4	25.2	9.0	3.3	7.3	13.1
Deformable DETR [17]	ResNet50	18.5	40.0	15.7	13.7	25.2	29.3	19.7	42.8	16.8	14.5	18.4	20.3
CE-SGNet (Ours)	ResNet50	**24.0**	**51.2**	**20.3**	**17.5**	**30.3**	**41.9**	**22.7**	**48.1**	**18.8**	**22.9**	**21.6**	23.4

**Table 2:** Ablation study of different models. Bold data denote the best performance.

Methods	ARFAM	SGRM	AP	AP_50_
Baseline			20.3	47.3
CE-SGNet (ours)	✓ ^1^		22.6	50.0
		✓	22.3	48.1
	✓	✓	**24.0**	**51.2**

^1^ ✓ signifies the utilization of this algorithm.

**Table 3:** Analysis of different attention mechanisms. Bold data denote the best performance.

Methods	GFLOPs	Params	AP	AP_50_
FPN	332.01	60.68 M	22.3	48.1
SENet	332.04	60.72 M	22.9	48.6
CAM	332.08	60.72 M	23.1	48.4
SAM	347.20	61.27 M	23.2	48.8
CBAM	332.09	60.72 M	23.3	49.9
ARFAM	347.27	61.31 M	**24.0 **	**51.2**

**Table 4:** Analysis of different positions of the ARFAM. Bold data denote the best performance.

Methods	GFLOPs	Params	AP	AP_50_
FPN	332.01	60.68 M	22.3	48.1
ARFAM+FPN	373.58	70.03 M	23.2	48.8
FPN+ARFAM-shared	347.27	60.80 M	23.1	49.0
FPN+ARFAM	347.27	61.31 M	**24.0**	**51.2**

**Table 5:** Analysis of different node embedding methods. Bold data denote the best performance.

Methods	GFLOPs	Params	AP	AP_50_
SGRM-hard	347.27	61.31 M	23.4	50.4
SGRM-soft	347.27	61.31 M	**24.0**	**51.2**

**Table 6:** Comparison results of different methods on the PCB dataset. Bold data denote the best performance. Underlined data denote second best performance.

Model	Backbone	AP	AP_50_	AP_75_	AP_*s*_	AP_*m*_	AP_*l*_
Faster RCNN [5]	ResNet50	52.8	96.3	50.3	35.5	52.7	47.1
Cascade RCNN [6]	ResNet50	53.1	96.7	53.0	34.2	53.2	49.3
RetinaNet [8]	ResNet50	52.8	95.8	52.4	20.0	53.0	47.8
FCOS [9]	ResNet50	53.0	96.3	52.3	26.6	53.6	44.5
ATSS [34]	ResNet50	53.4	**97.2**	**53.3**	26.7	53.6	50.2
GFL [32]	ResNet50	53.1	**97.2**	52.6	36.7	53.2	46.3
YOLOv5-s [33]	CSPDarkNet53	49.9	96.6	43.7	30.3	50.3	47.2
DETR [16]	ResNet50	36.9	87.6	22.4	4.5	37.4	36.8
Deformable DETR [17]	ResNet50	40.3	89.4	25.2	13.3	40.8	40.6
CE-SGNet (Ours)	ResNet50	**53.8**	97.0	52.7	**40.0**	**53.7**	**50.8**

**Table 7:** Comparsion results of different models on the complexity and time efficiency.

Model	GFLOPs	Params	FPS
Faster RCNN [5]	303.84	41.15 M	16.1
Cascade RCNN [6]	331.63	68.94 M	14.1
RetinaNet [8]	311.23	36.23 M	16.3
FCOS [9]	296.20	31.85 M	21.3
ATSS [34]	311.23	36.23 M	16.3
GFL [32]	307.94	32.05 M	18.4
YOLOv5-s [33]	43.87	7.04 M	54.2
DETR [16]	137.44	41.28 M	14.3
Deformable DETR [17]	292.81	39.82 M	9.6
CE-SGNet (Ours)	347.27	61.31 M	7.9

## Data Availability

The data presented in this study are available on request from the corresponding author.
